# 8-Methyl-2-[4-(trifluoro­meth­yl)phen­yl]-8*H*-pyrazolo­[4,3-*e*][1,2,4]triazolo[1,5-*c*]pyrimidin-5-amine methanol disolvate

**DOI:** 10.1107/S1600536810024591

**Published:** 2010-06-26

**Authors:** Anton V. Dolzhenko, Geok Kheng Tan, Anna V. Dolzhenko, Lip Lin Koh, Giorgia Pastorin

**Affiliations:** a Department of Pharmacy, Faculty of Science, National University of Singapore, 18 Science Drive 4, Singapore 117543, Singapore; b Department of Chemistry, Faculty of Science, National University of Singapore, 3 Science Drive 3, Singapore 117543, Singapore; c Perm State Pharmaceutical Academy, 2 Polevaya Street, Perm 614990, Russian Federation

## Abstract

In the title compound, C_14_H_10_F_3_N_7_·2CH_4_O, the heterocyclic ring system is essentially planar (r.m.s. deviation = 0.009 Å) and makes a dihedral angle of 6.91 (8)° with the attached benzene ring. In the crystal, the main mol­ecules form centrosymmetric *R*_2_^2^(8) dimers *via* pairs of N—H⋯N hydrogen bonds between the amino groups and pyrimidine N atoms. One of the independent methanol mol­ecules and its inversion equivalent are linked to the dimers *via* O—H⋯N and N—H⋯O hydrogen bonds, forming *R*_4_^4^(16) graph-set motifs. The dimers along with the hydrogen-bonded methanol mol­ecules are stacked along the *a* axis, with π–π inter­actions between the pyrazole and triazole rings [centroid–centroid distance = 3.4953 (10) Å].

## Related literature

For reviews on pyrazolo­[4,3-*e*][1,2,4]triazolo[1,5-*c*]pyrimidine adenosine receptor antagonists, see: Baraldi *et al.* (2006 ▶); Cacciari *et al.* (2007 ▶). For the general method used for the synthesis of the title compound, see: Dolzhenko *et al.* (2009 ▶); Cheong *et al.* (2010 ▶). For the crystal structures of related pyrazolo­[4,3-*e*][1,2,4]triazolo[1,5-*c*]pyrimidines, see: Ferretti *et al.* (2006 ▶); Mezheritsky *et al.* (2004 ▶); Tyurin *et al.* (2005 ▶); Xiao & Shi (2007 ▶). For graph-set analysis of hydrogen bonding, see: Bernstein *et al.* (1995 ▶).
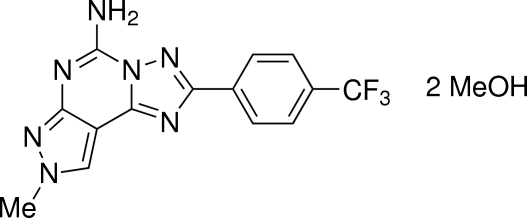


## Experimental

### 

#### Crystal data

C_14_H_10_F_3_N_7_·2CH_4_O
*M* *_r_* = 397.37
Monoclinic, 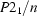

*a* = 4.6179 (3) Å
*b* = 17.1149 (10) Å
*c* = 22.7627 (13) Å
β = 94.323 (1)°
*V* = 1793.93 (19) Å^3^
*Z* = 4
Mo *K*α radiation
μ = 0.12 mm^−1^
*T* = 223 K
0.58 × 0.32 × 0.12 mm

#### Data collection

Bruker SMART APEX CCD diffractometer
Absorption correction: multi-scan (*SADABS*; Sheldrick, 2001 ▶) *T*_min_ = 0.932, *T*_max_ = 0.985
12385 measured reflections
4076 independent reflections
3538 reflections with *I* > 2σ(*I*)
*R*_int_ = 0.027

#### Refinement

*R*[*F* ^2^ > 2σ(*F* ^2^)] = 0.054
*wR*(*F* ^2^) = 0.145
*S* = 1.05
4076 reflections
266 parameters
H atoms treated by a mixture of independent and constrained refinement
Δρ_max_ = 0.35 e Å^−3^
Δρ_min_ = −0.21 e Å^−3^

### 

Data collection: *SMART* (Bruker, 2001 ▶); cell refinement: *SAINT* (Bruker, 2001 ▶); data reduction: *SAINT*; program(s) used to solve structure: *SHELXS97* (Sheldrick, 2008 ▶); program(s) used to refine structure: *SHELXL97* (Sheldrick, 2008 ▶); molecular graphics: *SHELXTL* (Sheldrick, 2008 ▶); software used to prepare material for publication: *SHELXTL*.

## Supplementary Material

Crystal structure: contains datablocks I, global. DOI: 10.1107/S1600536810024591/ci5111sup1.cif

Structure factors: contains datablocks I. DOI: 10.1107/S1600536810024591/ci5111Isup2.hkl

Additional supplementary materials:  crystallographic information; 3D view; checkCIF report

## Figures and Tables

**Table 1:** Hydrogen-bond geometry (Å, °)

*D*—H⋯*A*	*D*—H	H⋯*A*	*D*⋯*A*	*D*—H⋯*A*
O1*S*—H1*S*⋯N2^i^	0.83	2.05	2.877 (2)	175
O2*S*—H2*S*⋯N6	0.83	2.04	2.853 (2)	165
N7—H7*A*⋯O1*S*	0.85 (2)	2.46 (2)	3.050 (2)	128 (2)
N7—H7*B*⋯N3^i^	0.89 (2)	2.09 (3)	2.979 (2)	179 (2)

Symmetry code: (i) 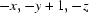.

**Fig. 1.**
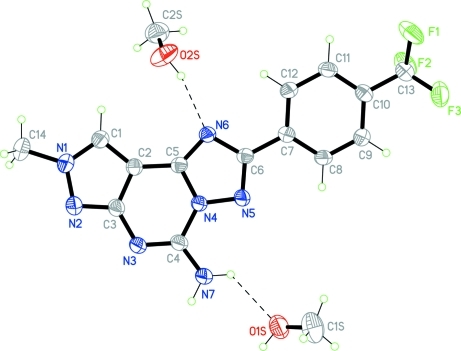
The asymmetric unit of the title compound, showing the atomic numbering scheme. Displacement ellipsoids are drawn at the 50% probability level.

**Fig. 2.**
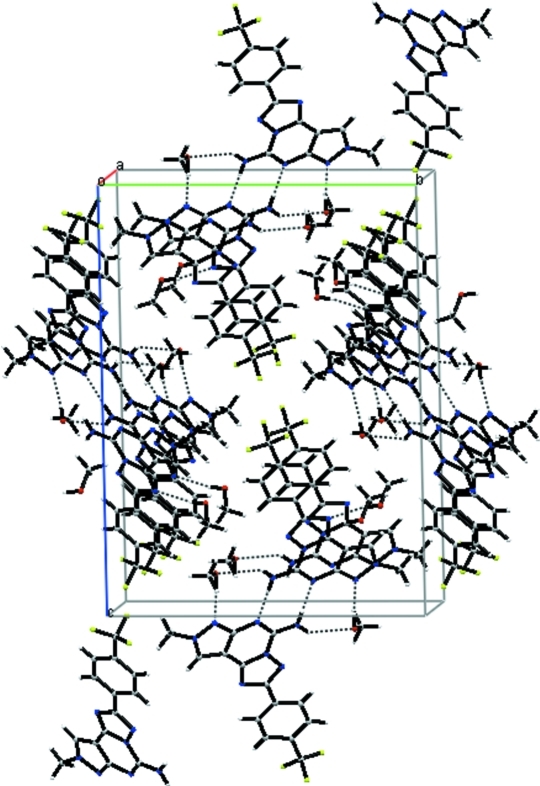
Crystal packing of the title compound, viewed along the a axis.

## supplementary crystallographic information

### Comment

Pyrazolo[4,3-*e*][1,2,4]triazolo[1,5-*c*]pyrimidine system has been recognized as an excellent template for the construction of new adenosine receptor antagonists (Baraldi *et al.*, 2006; Cacciari *et al.*, 2007). However, information on the structure of this heterocyclic system is limited (Ferretti *et al.*, 2006; Mezheritsky *et al.*, 2004; Tyurin *et al.*, 2005; Xiao & Shi, 2007). In continuation of our works on the development of new pyrazolo[4,3-*e*][1,2,4]triazolo[1,5-*c*]pyrimidine adenosine receptor antagonists (Dolzhenko *et al.*, 2009; Cheong *et al.*, 2010), we report here the molecular and crystal structure of 8-methyl-2-(4-trifluoromethylphenyl)-8*H*-pyrazolo[4,3-*e*][1,2,4]triazolo[1,5-*c*]pyrimidin-5-amine.

The compound crystallizes with two methanol solvent molecules. The heterocyclic ring system is essentially planar with an r.m.s. deviation of 0.009 Å. The phenyl ring makes a dihedral angle of 6.91 (8)° with the pyrazolo[4,3-*e*][1,2,4]triazolo[1,5-*c*]pyrimidine core. The trifluoromethyl group C atom, C13, is located 0.130 (3) Å above the C7—C12 mean plane.

In the crystal, molecules of 8-methyl-2-(4-trifluoromethylphenyl)-8*H*-pyrazolo[4,3-*e*][1,2,4]triazolo[1,5-*c*]pyrimidin-5-amine form centrosymmetric inversion dimers (Fig. 2). The pyrimidine N3 atom is connected with amino group N7—H7B of the pair molecule by intermolecular N···H—N hydrogen bond making *R*_2_^2^(8) graph-set motif (Bernstein *et al.*,1995). Methanol hydroxy group O1S—H1S also links the heterocyclic molecules in the dimer by the N—H···O—H···N hydrogen bond array with amino group N7—H7A and N2 of the pyrazole ring making *R*_4_^4^(16) graph-set motif. Another methanol molecule forms the O—H···N hydrogen bond with N6 of the triazole ring. The dimers are stacked along the *a* axis, with π–π interactions between pyrazole and triazole rings [centroid-to-centroid distance = 3.4953 (10) Å] (Fig. 2).

### Experimental

8-Methyl-2-(4-trifluoromethylphenyl)-8*H*-pyrazolo[4,3-*e*][1,2,4]triazolo][1,5-*c*]pyrimidin-5-amine was prepared from 8-methyl-2-(4-trifluoromethylphenyl)-8*H*-pyrazolo[4,3-*e*][1,2,4]triazolo][1,5-*c*]pyrimidine (Dolzhenko *et al.*, 2009) similarly to the described method (Cheong *et al.*, 2010). The detail procedure will be reported elsewhere. The crystals suitable for crystallographic analysis were grown by recrystallization from methanol. m.p. 573 K.

### Refinement

All C-bound H atoms were positioned geometrically and included in the refinement in riding-motion approximation [0.94 Å for CH of aromatic systems, 0.97 Å for methyl groups, and 0.83 Å for hydroxyl groups; *U*_iso_(H) = 1.2*U*_eq_(C_Ar_) and *U*_iso_(H) = 1.5*U*_eq_(O,C_Me_)] while the amino group H atoms were located in a difference map and refined freely.

### Crystal data

**Table:**

C_14_H_10_F_3_N_7_·2CH_4_O	*F*(000) = 824
*M**_r_* = 397.37	*D*_x_ = 1.471 Mg m^−^^3^
Monoclinic, *P*2_1_/*n*	Melting point: 573 K
Hall symbol: -P 2yn	Mo *K*α radiation, λ = 0.71073 Å
*a* = 4.6179 (3) Å	Cell parameters from 4421 reflections
*b* = 17.1149 (10) Å	θ = 2.4–27.2°
*c* = 22.7627 (13) Å	µ = 0.12 mm^−^^1^
β = 94.323 (1)°	*T* = 223 K
*V* = 1793.93 (19) Å^3^	Block, colourless
*Z* = 4	0.58 × 0.32 × 0.12 mm

### Data collection

**Table:**

Bruker SMART APEX CCD diffractometer	4076 independent reflections
Radiation source: fine-focus sealed tube	3538 reflections with *I* > 2σ(*I*)
graphite	*R*_int_ = 0.027
φ and ω scans	θ_max_ = 27.5°, θ_min_ = 1.5°
Absorption correction: multi-scan (*SADABS*; Sheldrick, 2001)	*h* = −5→5
*T*_min_ = 0.932, *T*_max_ = 0.985	*k* = −21→22
12385 measured reflections	*l* = −23→29

### Refinement

**Table:**

Refinement on *F*^2^	Primary atom site location: structure-invariant direct methods
Least-squares matrix: full	Secondary atom site location: difference Fourier map
*R*[*F*^2^ > 2σ(*F*^2^)] = 0.054	Hydrogen site location: inferred from neighbouring sites
*wR*(*F*^2^) = 0.145	H atoms treated by a mixture of independent and constrained refinement
*S* = 1.05	*w* = 1/[σ^2^(*F*_o_^2^) + (0.0746*P*)^2^ + 0.7421*P*] where *P* = (*F*_o_^2^ + 2*F*_c_^2^)/3
4076 reflections	(Δ/σ)_max_ = 0.001
266 parameters	Δρ_max_ = 0.35 e Å^−^^3^
0 restraints	Δρ_min_ = −0.21 e Å^−^^3^

### Special details

**Table:**

Geometry. All e.s.d.'s (except the e.s.d. in the dihedral angle between two l.s. planes) are estimated using the full covariance matrix. The cell e.s.d.'s are taken into account individually in the estimation of e.s.d.'s in distances, angles and torsion angles; correlations between e.s.d.'s in cell parameters are only used when they are defined by crystal symmetry. An approximate (isotropic) treatment of cell e.s.d.'s is used for estimating e.s.d.'s involving l.s. planes.
Refinement. Refinement of *F*^2^ against ALL reflections. The weighted *R*-factor *wR* and goodness of fit *S* are based on *F*^2^, conventional *R*-factors *R* are based on *F*, with *F* set to zero for negative *F*^2^. The threshold expression of *F*^2^ > 2σ(*F*^2^) is used only for calculating *R*-factors(gt) *etc*. and is not relevant to the choice of reflections for refinement. *R*-factors based on *F*^2^ are statistically about twice as large as those based on *F*, and *R*- factors based on ALL data will be even larger.

### Fractional atomic coordinates and isotropic or equivalent isotropic displacement parameters (Å^2^)

**Table:**

	*x*	*y*	*z*	*U*_iso_*/*U*_eq_	
O1S	0.5063 (3)	0.68580 (11)	0.08136 (7)	0.0581 (4)	
H1S	0.4507	0.6882	0.0459	0.087*	
C1S	0.8079 (5)	0.68768 (18)	0.08766 (13)	0.0684 (7)	
H1S1	0.8735	0.7415	0.0887	0.103*	
H1S2	0.8852	0.6611	0.0546	0.103*	
H1S3	0.8756	0.6617	0.1240	0.103*	
C2S	0.1594 (6)	0.15803 (13)	0.27649 (11)	0.0581 (6)	
H2S1	0.2028	0.1917	0.3103	0.087*	
H2S2	0.1779	0.1038	0.2887	0.087*	
H2S3	−0.0374	0.1678	0.2602	0.087*	
F1	1.4952 (3)	0.39342 (8)	0.43457 (6)	0.0616 (4)	
F2	1.2039 (3)	0.48312 (8)	0.45616 (5)	0.0550 (4)	
F3	1.5562 (3)	0.50993 (9)	0.40469 (6)	0.0642 (4)	
N1	−0.3122 (3)	0.23144 (9)	0.07561 (7)	0.0362 (3)	
N2	−0.2997 (3)	0.29563 (9)	0.04026 (7)	0.0375 (4)	
N3	−0.0193 (3)	0.41222 (8)	0.04749 (6)	0.0365 (4)	
N4	0.3019 (3)	0.41895 (8)	0.13254 (6)	0.0296 (3)	
N5	0.5146 (3)	0.45421 (8)	0.16847 (6)	0.0309 (3)	
N6	0.3934 (3)	0.33542 (8)	0.20487 (6)	0.0286 (3)	
N7	0.2815 (4)	0.51915 (10)	0.06445 (8)	0.0461 (4)	
H7A	0.402 (5)	0.5454 (13)	0.0863 (10)	0.044 (6)*	
H7B	0.204 (5)	0.5402 (14)	0.0312 (11)	0.052 (6)*	
C1	−0.1317 (4)	0.23497 (10)	0.12400 (8)	0.0343 (4)	
H1	−0.1081	0.1971	0.1539	0.041*	
C2	0.0145 (4)	0.30557 (9)	0.12153 (7)	0.0301 (4)	
C3	−0.0985 (4)	0.34097 (10)	0.06864 (7)	0.0322 (4)	
C4	0.1813 (4)	0.45034 (10)	0.07964 (7)	0.0336 (4)	
C5	0.2331 (4)	0.34778 (9)	0.15530 (7)	0.0276 (3)	
C6	0.5597 (4)	0.40191 (9)	0.21105 (7)	0.0269 (3)	
C7	0.7738 (3)	0.41460 (9)	0.26155 (7)	0.0274 (3)	
C8	0.9154 (4)	0.48630 (10)	0.26875 (7)	0.0326 (4)	
H8	0.8786	0.5261	0.2407	0.039*	
C9	1.1098 (4)	0.49892 (10)	0.31706 (8)	0.0343 (4)	
H9	1.2041	0.5474	0.3221	0.041*	
C10	1.1650 (4)	0.43965 (10)	0.35808 (7)	0.0307 (4)	
C11	1.0299 (4)	0.36786 (10)	0.35067 (8)	0.0343 (4)	
H11	1.0710	0.3277	0.3782	0.041*	
C12	0.8337 (4)	0.35550 (10)	0.30243 (8)	0.0331 (4)	
H12	0.7407	0.3068	0.2974	0.040*	
C13	1.3561 (4)	0.45625 (11)	0.41268 (8)	0.0368 (4)	
C14	−0.5043 (5)	0.16731 (12)	0.05670 (10)	0.0464 (5)	
H14A	−0.4241	0.1396	0.0245	0.070*	
H14B	−0.6940	0.1879	0.0436	0.070*	
H14C	−0.5229	0.1317	0.0894	0.070*	
O2S	0.3553 (4)	0.17366 (9)	0.23347 (8)	0.0626 (5)	
H2S	0.3819	0.2215	0.2314	0.094*	

### Atomic displacement parameters (Å^2^)

**Table:**

	*U*^11^	*U*^22^	*U*^33^	*U*^12^	*U*^13^	*U*^23^
O1S	0.0507 (9)	0.0792 (12)	0.0434 (8)	−0.0125 (8)	−0.0029 (7)	−0.0038 (8)
C1S	0.0505 (14)	0.0805 (18)	0.0727 (17)	0.0068 (12)	−0.0050 (12)	−0.0207 (14)
C2S	0.0740 (16)	0.0445 (12)	0.0554 (13)	0.0039 (11)	0.0027 (12)	0.0023 (10)
F1	0.0664 (9)	0.0601 (8)	0.0537 (8)	0.0231 (7)	−0.0252 (6)	−0.0033 (6)
F2	0.0506 (7)	0.0806 (9)	0.0333 (6)	0.0123 (6)	0.0006 (5)	−0.0145 (6)
F3	0.0544 (8)	0.0857 (10)	0.0502 (7)	−0.0294 (7)	−0.0117 (6)	0.0062 (7)
N1	0.0395 (8)	0.0307 (8)	0.0381 (8)	−0.0050 (6)	0.0010 (6)	−0.0035 (6)
N2	0.0428 (9)	0.0336 (8)	0.0348 (8)	−0.0033 (6)	−0.0047 (6)	−0.0025 (6)
N3	0.0491 (9)	0.0286 (7)	0.0300 (7)	−0.0014 (6)	−0.0081 (6)	0.0023 (6)
N4	0.0391 (8)	0.0237 (7)	0.0254 (7)	−0.0008 (5)	−0.0027 (5)	−0.0003 (5)
N5	0.0385 (8)	0.0260 (7)	0.0273 (7)	−0.0014 (6)	−0.0036 (6)	−0.0018 (5)
N6	0.0343 (7)	0.0250 (7)	0.0264 (7)	0.0006 (5)	0.0011 (5)	0.0003 (5)
N7	0.0665 (12)	0.0316 (8)	0.0369 (9)	−0.0117 (8)	−0.0177 (8)	0.0086 (7)
C1	0.0389 (9)	0.0310 (9)	0.0332 (9)	−0.0026 (7)	0.0030 (7)	−0.0004 (7)
C2	0.0350 (9)	0.0278 (8)	0.0274 (8)	0.0001 (6)	0.0019 (6)	−0.0020 (6)
C3	0.0382 (9)	0.0286 (8)	0.0292 (8)	0.0018 (7)	−0.0014 (7)	−0.0029 (6)
C4	0.0441 (10)	0.0277 (8)	0.0279 (8)	0.0018 (7)	−0.0037 (7)	0.0018 (6)
C5	0.0335 (8)	0.0232 (7)	0.0264 (7)	0.0026 (6)	0.0040 (6)	−0.0013 (6)
C6	0.0322 (8)	0.0234 (7)	0.0253 (7)	0.0024 (6)	0.0031 (6)	−0.0008 (6)
C7	0.0295 (8)	0.0277 (8)	0.0252 (7)	0.0032 (6)	0.0029 (6)	−0.0008 (6)
C8	0.0402 (9)	0.0272 (8)	0.0299 (8)	−0.0004 (7)	−0.0010 (7)	0.0052 (6)
C9	0.0374 (9)	0.0300 (8)	0.0349 (9)	−0.0041 (7)	−0.0011 (7)	−0.0002 (7)
C10	0.0281 (8)	0.0361 (9)	0.0279 (8)	0.0051 (7)	0.0022 (6)	−0.0005 (7)
C11	0.0398 (9)	0.0312 (9)	0.0314 (8)	0.0042 (7)	−0.0016 (7)	0.0068 (7)
C12	0.0394 (9)	0.0252 (8)	0.0344 (9)	−0.0010 (7)	0.0000 (7)	0.0021 (6)
C13	0.0350 (9)	0.0426 (10)	0.0323 (9)	0.0037 (7)	0.0002 (7)	−0.0005 (7)
C14	0.0476 (11)	0.0386 (10)	0.0526 (12)	−0.0119 (9)	0.0009 (9)	−0.0091 (8)
O2S	0.0839 (12)	0.0294 (7)	0.0764 (11)	0.0002 (8)	0.0186 (9)	0.0034 (7)

### Geometric parameters (Å, °)

**Table:**

O1S—C1S	1.390 (3)	N7—C4	1.321 (2)
O1S—H1S	0.83	N7—H7A	0.85 (2)
C1S—H1S1	0.97	N7—H7B	0.89 (2)
C1S—H1S2	0.97	C1—C2	1.387 (2)
C1S—H1S3	0.97	C1—H1	0.94
C2S—O2S	1.408 (3)	C2—C3	1.412 (2)
C2S—H2S1	0.97	C2—C5	1.419 (2)
C2S—H2S2	0.97	C6—C7	1.475 (2)
C2S—H2S3	0.97	C7—C12	1.388 (2)
F1—C13	1.330 (2)	C7—C8	1.394 (2)
F2—C13	1.338 (2)	C8—C9	1.383 (2)
F3—C13	1.325 (2)	C8—H8	0.94
N1—C1	1.331 (2)	C9—C10	1.389 (2)
N1—N2	1.366 (2)	C9—H9	0.94
N1—C14	1.456 (2)	C10—C11	1.382 (3)
N2—C3	1.339 (2)	C10—C13	1.496 (2)
N3—C4	1.310 (2)	C11—C12	1.386 (2)
N3—C3	1.371 (2)	C11—H11	0.94
N4—N5	1.3698 (19)	C12—H12	0.94
N4—C5	1.370 (2)	C14—H14A	0.97
N4—C4	1.396 (2)	C14—H14B	0.97
N5—C6	1.324 (2)	C14—H14C	0.97
N6—C5	1.319 (2)	O2S—H2S	0.83
N6—C6	1.374 (2)		
			
C1S—O1S—H1S	109.5	N6—C5—N4	109.56 (14)
O1S—C1S—H1S1	109.5	N6—C5—C2	135.39 (15)
O1S—C1S—H1S2	109.5	N4—C5—C2	115.05 (14)
H1S1—C1S—H1S2	109.5	N5—C6—N6	115.41 (14)
O1S—C1S—H1S3	109.5	N5—C6—C7	122.01 (14)
H1S1—C1S—H1S3	109.5	N6—C6—C7	122.58 (14)
H1S2—C1S—H1S3	109.5	C12—C7—C8	119.65 (15)
O2S—C2S—H2S1	109.5	C12—C7—C6	120.21 (15)
O2S—C2S—H2S2	109.5	C8—C7—C6	120.14 (14)
H2S1—C2S—H2S2	109.5	C9—C8—C7	120.09 (15)
O2S—C2S—H2S3	109.5	C9—C8—H8	120.0
H2S1—C2S—H2S3	109.5	C7—C8—H8	120.0
H2S2—C2S—H2S3	109.5	C8—C9—C10	119.71 (16)
C1—N1—N2	113.54 (14)	C8—C9—H9	120.1
C1—N1—C14	127.47 (16)	C10—C9—H9	120.1
N2—N1—C14	118.95 (15)	C11—C10—C9	120.58 (16)
C3—N2—N1	103.90 (14)	C11—C10—C13	120.16 (16)
C4—N3—C3	116.28 (14)	C9—C10—C13	119.11 (16)
N5—N4—C5	110.01 (13)	C10—C11—C12	119.62 (15)
N5—N4—C4	124.55 (14)	C10—C11—H11	120.2
C5—N4—C4	125.41 (14)	C12—C11—H11	120.2
C6—N5—N4	101.84 (13)	C11—C12—C7	120.34 (16)
C5—N6—C6	103.18 (13)	C11—C12—H12	119.8
C4—N7—H7A	123.1 (15)	C7—C12—H12	119.8
C4—N7—H7B	117.2 (15)	F3—C13—F1	106.84 (16)
H7A—N7—H7B	119 (2)	F3—C13—F2	105.89 (16)
N1—C1—C2	106.40 (15)	F1—C13—F2	105.45 (15)
N1—C1—H1	126.8	F3—C13—C10	113.04 (15)
C2—C1—H1	126.8	F1—C13—C10	113.31 (15)
C1—C2—C3	104.97 (15)	F2—C13—C10	111.71 (14)
C1—C2—C5	138.58 (16)	N1—C14—H14A	109.5
C3—C2—C5	116.45 (15)	N1—C14—H14B	109.5
N2—C3—N3	122.67 (15)	H14A—C14—H14B	109.5
N2—C3—C2	111.18 (15)	N1—C14—H14C	109.5
N3—C3—C2	126.15 (15)	H14A—C14—H14C	109.5
N3—C4—N7	122.99 (16)	H14B—C14—H14C	109.5
N3—C4—N4	120.64 (15)	C2S—O2S—H2S	109.5
N7—C4—N4	116.37 (16)		
			
C1—N1—N2—C3	0.3 (2)	C1—C2—C5—N6	0.6 (4)
C14—N1—N2—C3	−177.53 (16)	C3—C2—C5—N6	−178.44 (18)
C5—N4—N5—C6	−0.34 (17)	C1—C2—C5—N4	−179.5 (2)
C4—N4—N5—C6	−178.42 (15)	C3—C2—C5—N4	1.4 (2)
N2—N1—C1—C2	−0.4 (2)	N4—N5—C6—N6	0.51 (18)
C14—N1—C1—C2	177.22 (17)	N4—N5—C6—C7	−179.41 (14)
N1—C1—C2—C3	0.29 (19)	C5—N6—C6—N5	−0.49 (19)
N1—C1—C2—C5	−178.9 (2)	C5—N6—C6—C7	179.44 (14)
N1—N2—C3—N3	179.97 (16)	N5—C6—C7—C12	−174.11 (16)
N1—N2—C3—C2	−0.1 (2)	N6—C6—C7—C12	6.0 (2)
C4—N3—C3—N2	−179.75 (17)	N5—C6—C7—C8	6.4 (2)
C4—N3—C3—C2	0.3 (3)	N6—C6—C7—C8	−173.47 (15)
C1—C2—C3—N2	−0.1 (2)	C12—C7—C8—C9	−1.4 (3)
C5—C2—C3—N2	179.25 (15)	C6—C7—C8—C9	178.09 (16)
C1—C2—C3—N3	179.81 (17)	C7—C8—C9—C10	0.5 (3)
C5—C2—C3—N3	−0.8 (3)	C8—C9—C10—C11	0.8 (3)
C3—N3—C4—N7	179.18 (18)	C8—C9—C10—C13	−174.85 (16)
C3—N3—C4—N4	−0.5 (3)	C9—C10—C11—C12	−1.2 (3)
N5—N4—C4—N3	179.14 (16)	C13—C10—C11—C12	174.37 (16)
C5—N4—C4—N3	1.3 (3)	C10—C11—C12—C7	0.4 (3)
N5—N4—C4—N7	−0.6 (3)	C8—C7—C12—C11	0.9 (3)
C5—N4—C4—N7	−178.36 (17)	C6—C7—C12—C11	−178.53 (16)
C6—N6—C5—N4	0.23 (17)	C11—C10—C13—F3	154.07 (17)
C6—N6—C5—C2	−179.89 (18)	C9—C10—C13—F3	−30.3 (2)
N5—N4—C5—N6	0.06 (19)	C11—C10—C13—F1	32.3 (2)
C4—N4—C5—N6	178.13 (15)	C9—C10—C13—F1	−152.04 (17)
N5—N4—C5—C2	−179.84 (14)	C11—C10—C13—F2	−86.6 (2)
C4—N4—C5—C2	−1.8 (2)	C9—C10—C13—F2	89.0 (2)

### Hydrogen-bond geometry (Å, °)

**Table:**

*D*—H···*A*	*D*—H	H···*A*	*D*···*A*	*D*—H···*A*
O1S—H1S···N2^i^	0.83	2.05	2.877 (2)	175
O2S—H2S···N6	0.83	2.04	2.853 (2)	165
N7—H7A···O1S	0.85 (2)	2.46 (2)	3.050 (2)	128 (2)
N7—H7B···N3^i^	0.89 (2)	2.09 (3)	2.979 (2)	179 (2)

Symmetry codes: (i) −*x*, −*y*+1, −*z*.
